# Inflammatory and oxidative stress airway markers in premature newborns of hypertensive mothers

**DOI:** 10.1590/1414-431X20165160

**Published:** 2016-08-01

**Authors:** R.J. Madoglio, L.M.S.S. Rugolo, C.S. Kurokawa, M.P.A. Sá, J.C. Lyra, L.C.O. Antunes

**Affiliations:** 1 Curso de Pós-Graduação de Ginecologia e Obstetrícia, Faculdade de Medicina de Botucatu, Universidade Estadual Paulista, Botucatu, SP, Brasil; 2 Departamento de Pediatria, Faculdade de Medicina de Botucatu, Universidade Estadual Paulista, Botucatu, SP, Brasil; 3 Divisão de Fisioterapia, Faculdade de Medicina de Botucatu, Universidade Estadual Paulista, Botucatu, SP, Brasil

**Keywords:** Infant, Premature, Pre-eclampsia, Oxidative stress, Cytokines, Bronchopulmonary dysplasia

## Abstract

Although oxidative stress and inflammation are important mechanisms in the pathophysiology of preeclampsia and preterm diseases, their contribution to the respiratory prognosis of premature infants of hypertensive mothers is not known. Our objective was to determine the levels of oxidative stress and inflammation markers in the airways of premature infants born to hypertensive and normotensive mothers, in the first 72 h of life, and to investigate whether they are predictors of bronchopulmonary dysplasia (BPD)/death. This was a prospective study with premature infants less than 34 weeks’ gestation on respiratory support who were stratified into 2 groups: 32 premature infants of hypertensive mothers and 41 of normotensive women, with a mean gestational age of 29 weeks. Exclusion criteria were as follows: diabetes mellitus, chorioamnionitis, malformation, congenital infection, and death within 24 h after birth. The outcome of interest was BPD/death. Malondialdehyde (MDA), nitric oxide (NO), and interleukin 8 (IL-8) were measured in airway aspirates from the first and third days of life and did not differ between the groups. Univariate and multivariate statistical analyses were performed. The concentrations of MDA, NO, and IL-8 were not predictors of BPD/death. Premature infants who developed BPD/death had higher levels of IL-8 in the first days of life. The gestational age, mechanical ventilation, and a small size for gestational age were risk factors for BPD/death. In conclusion, the biomarkers evaluated were not increased in premature infants of hypertensive mothers and were not predictors of BPD/death.

## Introduction

Hypertensive disorders of pregnancy are the most common medical complications of pregnancy and are an important cause of maternal, fetal, and neonatal morbidity and mortality. The consequences of these disorders in the infant most notably include intrauterine growth restriction, prematurity, and increased risk of neonatal diseases (1).

Preeclampsia, the most common manifestation of hypertensive disorders, is attributed to inadequate placentation and reduced uteroplacental blood flow, causing ischemia/reperfusion and release of various mediators involved in the pathophysiology of the disease, including cytokines and products of oxidative stress (1). In pregnancies complicated by hypertensive disorders, the levels of pro-oxidant agents are increased and the levels of antioxidants are decreased in the maternal blood and placenta (2,3). A limited number of studies have been carried out on fetuses and newborns, suggesting that infants born from preeclamptic mothers are exposed to increased oxidative stress (4). However, the relationship between hypertensive disorders of pregnancy, oxidative stress, and neonatal prognosis has been scarcely studied and the results are inconclusive.

Hypertensive disorders of pregnancy are a leading cause of premature births and some studies have shown an increased risk of acute and chronic lung disease in premature infants born to preeclamptic mothers. Furthermore, there is increasing evidence suggesting that inflammatory mediators and oxidative stress may be implicated in the pathogenesis of several diseases in premature infants (5).

Bronchopulmonary dysplasia (BPD) is the main pulmonary complication in premature infants exposed to multiple risk factors including mechanical ventilation, oxygen use, *patent ductus arteriosus*, inflammation, and infection (6,7). Elevated levels of lipid and protein oxidation products and pro-inflammatory cytokines in premature infants during the first days of life have been associated with adverse lung development, suggesting the involvement of oxidative stress and inflammatory mediators in the pathogenesis of BPD. However, an ideal marker for predicting the risk of BPD has not yet been identified (8–10).

The aim of this study was to investigate whether the levels of oxidative stress and inflammation markers are elevated in the airways of premature infants of hypertensive mothers during the first 72 h after birth and whether they are predictors of BPD/death.

## Material and Methods

This was an observational, prospective, and longitudinal study involving premature infants born before 34 weeks of gestation and admitted to the Neonatal Intensive Care Unit of the Faculdade de Medicina de Botucatu, Universidade Estadual Paulista, from March 2009 until September 2010. The study was approved by local Ethics Committee and written informed consent was obtained for each case.

Pregnant women with premature labor or medically indicated preterm delivery before 34 weeks, without diabetes mellitus and chorioamnionitis were selected.

Hypertensive disorders of pregnancy were defined according to the National High Blood Pressure Education Program (11), which used the blood pressure level of 140/90 mmHg or higher on 2 separate occasions at least 4 h apart as diagnostic criteria for hypertension. Preeclampsia was characterized as hypertension manifested after 20 weeks of gestation, associated with proteinuria (≥0.3 g in a 24-h urine specimen), in previously normotensive women (11).

Premature infants with a gestational age less than 34 weeks and birth weight less than 1800 g, who underwent mechanical ventilation in the first 24 h after birth, were included. Neonates with congenital malformations and/or congenital infections, and those who died in the first 24 h, were excluded.

### Airway biological material samples

A 1.5-mL sample of airway aspirate was obtained by tracheal aspiration in intubated newborns and nasopharyngeal aspiration in those with nasal continuous positive airway pressure (CPAP). The procedure lasted 15 s, under rigorous monitoring of the babies.

Tracheal aspirate was collected in a standardized way, by the same professional at 2 different times: around 24 h after birth and on the third day of life. Preterm babies who were treated with surfactant had the first sample collected >6 h after surfactant administration. To collect the sample, 1 mL of sterile 0.9% saline was instilled using a syringe via a 5-F gauge tube feeding catheter that had been placed through the tracheal tube and advanced to the tip or through the nostrils in newborns on nasal CPAP. The saline was instilled and immediately drawn back into the syringe (12,13). The recovered volume of biological material obtained in the procedure was 85% on average. After collection, the catheter was washed with 1 mL of 0.9% saline and the collected material was transferred from the syringe into cryotubes, immediately frozen in liquid nitrogen and stored at -80°C until the time of analysis.

### Methods of biochemical analysis

Malondialdehyde (MDA) and nitric oxide (NO) were measured as markers of oxidative stress and interleukin-8 (IL-8) was measured as an inflammatory marker.

The MDA concentration was determined by spectrophotometry using the TBARS assay kit from Cayman Chemical Company^¯^ (USA). The TBARS is a classical method for identifying products of lipid peroxidation, especially MDA, as described by Ohkawa et al. in 1979 (14). The intra-assay accuracy of the method is 5.5–7.6% and inter-assay accuracy is 5.1–5.9%, with a lower limit of detection of 0.15 μM.

The NO concentration was determined by a colorimetric method using a Nitrate/Nitrite colorimetric assay kit (Cayman Chemical Company^¯^). The amount of NO was determined by the measurement of nitrate and nitrite after treatment with nitrate reductase according to the Griess method (15). This method has good accuracy, with an inter-assay coefficient of variation of 3.4%, an intra-assay coefficient of variation of 2.7%, and a minimum detection limit of 2.0 μM.

Concentrations of IL-8 were measured by an enzyme-linked immunosorbent assay (ELISA) using the Duo Set to human IL-8 kit. Reagents were supplied by BD Bioscience Pharmingen (USA) and were used according to the supplier's instructions. The lower limit of detection of IL-8 was 3.5 pg/mL, with an inter-assay coefficient of variation of 8% and an intra-assay coefficient of variation of 5%.

### Study variables

The maternal and gestational data included the following: age, parity, presence or absence of hypertension, use of antenatal corticosteroids (≥1 dose), premature rupture of membranes ≥12 h, premature labor, fetal distress, and type of delivery. The severity of preeclampsia was characterized by the occurrence of hemolysis or eclampsia, elevated liver enzymes, low platelets (HELLP) syndrome.

Newborns were evaluated for the following variables: gender; gestational age (determined by the best obstetric estimate); birth weight (g), and appropriate weight for gestational age according to the criteria of Alexander et al. (16); resuscitation in the delivery room, which was characterized by the need for positive pressure ventilation; APGAR score in the first and fifth min; neonatal morbidity, including the presence of acute respiratory disease and BPD (oxygen requirement for at least 28 days, plus assessment of disease severity at 36 weeks post-menstrual age) (17), and sepsis (clinical or confirmed, defined as early-onset if diagnosed within the first 72 h of life and late-onset, if after 72 h). We also evaluated surfactant use, the need for conventional mechanical ventilation or nasal CPAP in the first 72 h of life, length of stay, and death.

The outcomes of interest were discharge without BPD or BPD/death. As death is a competing outcome for BPD, we used the composite outcome BPD/death including infants with BPD who survived or died, and also those on use of oxygen who died before 28 days of life, so the diagnosis of BPD could not be done.

### Statistical analysis

The data are reported as the number and percent of events, the mean and standard deviation, or the median and percentiles.

The comparison of numerical variables between groups was performed using Student's *t*-test or the Mann-Whitney test and the comparison of categorical variables was performed using the chi-square or the Fisher's exact test.

To test the association between hypertensive pregnancy disorders and the levels of biomarkers and to assess the relationship of biomarkers with the occurrence of BPD and/or death, generalized linear models with gamma distribution were adjusted by PROCGENMOD using the program SAS v.9.2 for Windows (SAS Institute Inc., USA).

Multivariate logistic regression analysis with a stepwise strategy was used to identify independent risk factors for BPD/death. Differences were considered to be significant when P<0.05.

## Results

Seventy-three pregnant women satisfied the inclusion criteria, 32 with hypertensive disorders of pregnancy and 41 normotensive. Among the normotensive group, 2 newborns were not included in the study due to the presence of maternal chorioamnionitis.

All hypertensive pregnant women had preeclampsia, and in 9 cases (28%) preeclampsia was superimposed on chronic hypertension. Preeclampsia was severe in 12 cases (6 progressed to eclampsia and 6 had HELLP syndrome). Magnesium sulfate was used to treat 8 pregnant women.

Emergency cesarean section was the main route of birth in the hypertensive group, whereas preterm labor predominated in the normotensive group. A significantly higher proportion of infants that were small for gestational age were observed in the hypertensive group. Most newborns required resuscitation in the delivery room and the Apgar scores at 1 and 5 min did not differ between the groups (Table 1).

**Table:**
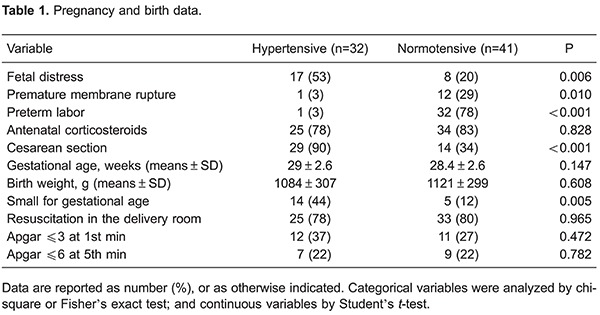

Overall 35% of this sample developed BPD (oxygen requirement for at least 28 days). Neonatal morbidity is summarized in Table 2.

**Table:**
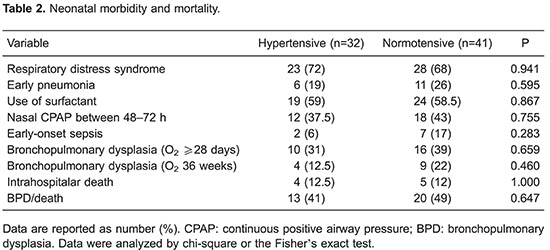

In newborns of hypertensive and normotensive mothers, the medians and percentiles of maximum fraction of inspired oxygen (FiO_2_) in the first 72 h were, respectively, 0.4 (0.3–0.5) and 0.5 (0.3–0.6) (P=0.225); duration of oxygen use was 7.5 (5–30) and 18 (7.5–52) days (P=0.064), and the length of hospitalization was 30 (21–53) and 42 (31–69) days (P=0.122).

The levels of oxidative stress markers (MDA and NO) and inflammation (IL-8) did not differ between premature infants of hypertensive and normotensive mothers on either day 1 or day 3 of ventilatory support. Furthermore, no significant difference was observed when the values of the biomarkers in the first versus the third day in each group were compared (Table 3).

**Table:**
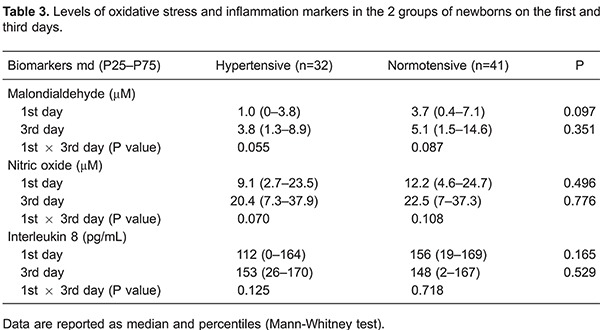

**Figure 1:**
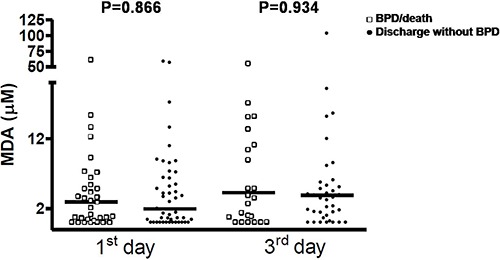
Malondialdehyde (MDA) levels in premature infants on the 1st and 3rd days of life according to the outcomes bronchopulmonary dysplasia (BPD)/death or discharge without BPD. Data are reported as median and percentiles (Mann-Whitney test).

**Figure 2:**
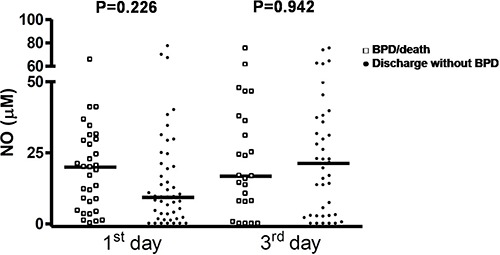
Nitric oxide (NO) levels in premature infants on the 1st and 3rd days of life according to the outcomes bronchopulmonary dysplasia (BPD)/death or discharge without BPD. Data are reported as median and percentiles (Mann-Whitney test).

**Figure 3:**
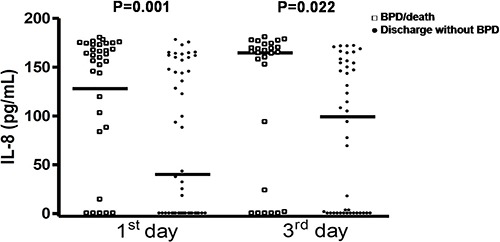
Levels of IL-8 in premature infants at the 1st and 3rd days of life according to the outcomes bronchopulmonary dysplasia (BPD)/death or discharge without BPD. Data are reported as median and percentiles (Mann-Whitney test).

The concentrations of MDA and NO in airways were not associated with the outcome BPD/death (Figures 1 and 2). However, IL-8 was significantly increased in the first and third days of life in premature infants who developed BPD/death (Figure 3).

The fit of generalized linear models with gamma distribution to test the association between hypertensive disorders of pregnancy, gestational age, birth weight appropriateness for gestational age, use of mechanical ventilation, and levels of biomarkers showed no significant association between these variables and the concentrations of biomarkers at both evaluation periods.

Using multivariate logistic regression, we included the following variables as potential risk factors for BPD/death: hypertensive disorders of pregnancy, the values of MDA, NO, and IL-8 at the 2 time-points, gestational age, mechanical ventilation, and variables that were significant in bivariate analysis. Among these, mechanical ventilation, gestational age, and small for gestational age were identified as independent risk factors for BPD/death (Table 4).

**Table:**
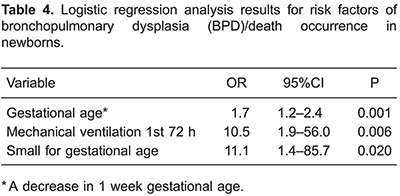

## Discussion

The impact of the inflammatory condition and the increased oxidative stress of preeclampsia on the newborn have not been established. Thus, the contribution of this study was to examine some aspects not yet studied in the literature: the levels of MDA, NO, and IL-8 in the airways of premature infants of mothers with preeclampsia. Our results showed that these markers did not increase in the first 72 h of life as a consequence of maternal disease.

Although bronchoalveolar lavage is considered the most accurate method for obtaining airway biological material, tracheal aspiration is the most widely used technique because it is a routine procedure, is less aggressive, and is standardized in neonatal care (10). This study confirmed the effectiveness of tracheal aspirate, with 85% recovery of the instilled volume.

Investigations regarding the association between preeclampsia and BPD have showed conflicting results. In a cohort of 107 premature infants with less than 32 weeks of gestation, preeclampsia was an independent risk factor for BPD, with an odds ratio (OR)=18.7 (95%CI=2.44–144.76) (18). On the other hand, in a large multicenter cohort of very low birth weight infants, maternal preeclampsia was associated with a reduced risk of BPD in infants with gestational age greater than 31 weeks and in small for gestational age preterm infants (19). However, in our study preeclampsia was not associated with the occurrence of BPD, as previously reported by Cetinkaya et al. (20).

Obstetric management of maternal hypertension is an important factor for neonatal prognosis, as demonstrated in a recent study of 1858 pregnant women with mild and stable gestational hypertension, in which 25.5% had iatrogenic elective late preterm delivery with increased neonatal respiratory morbidity (21). Thus, it is still unclear whether the increased adverse perinatal outcome in infants of hypertensive mothers is due to shorter gestation or if there is a direct effect of maternal disease and/or treatment on the fetus.

While the involvement of oxidative stress in the pathophysiology of BPD has been reported, few studies have investigated the levels of oxidative stress markers and antioxidants in the airways of premature infants who developed BPD.

In our study, MDA and NO levels in airways were not associated with occurrence of BPD/death, whereas levels of IL-8 were significantly higher in premature infants who developed BPD/death. Schock et al. (8) assayed protein oxidation products and antioxidant agents in the broncho-alveolar lavage samples of premature infants during the first weeks of life and found increased levels of antioxidants within the first 3 days in those that developed BPD (O_2_ at 36 weeks post-menstrual age). However, the oxidative stress markers did not differ in relation to premature infants without BPD.

Elevated levels of MDA and reduced glutathione were reported in the airways from premature infants of less than 32 weeks who were ventilated in the first week and developed BPD; however, the predictors of BPD were tracheal infection, sepsis, and gestational age (9).

Vyas et al. (12) evaluated weekly NO levels in the bronchoalveolar lavage fluid of 12 premature infants who developed BPD (O_2_ at 28 days of age), 18 with respiratory distress syndrome and without BPD and 7 full-term newborns without lung disease (control). Although a difference was not observed between the groups in the first week, the NO levels remained elevated in premature infants who developed BPD and decreased in the other groups from the second week, reaching a significant difference at 14 days of life. The authors suggested that a high initial level of NO may be an important mediator for adapting pulmonary circulation after birth, whereas the persistence of elevated levels in premature infants with BPD could be due to early maladaptation of pulmonary circulation or ongoing pulmonary inflammation in the second week of life. This postnatal variation in NO levels may explain the results of our study because we evaluated NO only in the first 72 h of life and found no differences between the groups. The NO levels we detected on day 3 were similar to those found in the above mentioned study in the first week, which is in accordance with the authors' hypothesis regarding the role of NO in the pulmonary circulatory adaptation during the first days of life.

As lung inflammation is an important contributor to the pathogenesis of BPD, many studies have attempted to identify early inflammatory biomarkers that could reflect lung injury and predict the development of BPD. A recent review highlighted the need for BPD predictors, and discussed the challenge of searching for a reliable predictor. Most clinical predictors have shown moderate predictive accuracy, and, despite various laboratory biomarkers being under investigation, none is currently used in clinical practice (22).

Several authors have reported an increase in pro-inflammatory cytokines, especially IL-8, in the airways of premature infants who develop BPD (10,13,23). In accordance with these data, we found that preterm infants with BPD/death had increased levels of IL-8 in tracheal aspirate on the first and 3rd day of life. However, IL-8 levels were not associated with the development of BPD/death after adjustment for clinical variables.

In a systematic review to evaluate early biomarkers as predictors for BPD, 16 studies were included and 21 biomarkers were investigated. Only one study assessed IL-8 in tracheal aspirate and showed that IL-8 levels were significantly higher in preterm infants who developed BPD than in those without BPD. A cut-off ≥400 ng/mL on days 1 and 3 had a sensitivity of 60 and 71%, respectively. In the systematic review, IL-8 was not identified as good predictor for BPD, and the authors concluded that there is not enough evidence to determine which biomarkers have clinical application in predicting BPD. Further studies are necessary (24).

A large multicenter study evaluated if serum cytokine patterns would be useful in predicting BPD or death in extremely low birth weight infants. Logistic regression models were developed using clinical and cytokine data. BPD/death was associated with higher IL-8 levels in the first days, male gender, low gestational age, small for gestational age and mechanical ventilation. However, the association of clinical variables with outcome was stronger than cytokine levels, and the authors suggested cytokines alone are unlikely to be useful in clinical practice (25).

Our results are in agreement with this multicenter study, highlighting the importance of clinical variables as predictors of BPD/death. We identified, by multivariate analysis, 3 risk factors for BPD/death: gestational age, small for gestational age, and the use of mechanical ventilation in the first days of life.

Intrauterine growth restriction is a common complication of hypertensive disorders of pregnancy, and a high incidence of small for gestational age infants in preeclamptic women has been reported, ranging from 15% to more than 50% (1). This was confirmed in our study, in which 44% of premature infants of hypertensive mothers were small for gestational age. Intrauterine growth restriction causes structural and functional lung abnormalities that are present at birth, during childhood, and can persist into adulthood (26). In this study, small for gestational age was an independent risk factor for BPD/death, which is in agreement with several previous studies that showed increased mortality and increased risk of BPD in preterm small for gestational age infants compared to those who were appropriate for gestational age (27,28).

The present study has some limitations: a small sample size, lack of a healthy term control group, and the postnatal age of sampling was restricted to the first 3 days of life. However, to our knowledge, this study is the first to report concentrations of oxidative stress markers and IL-8 in the airway of premature infants born to hypertensive mothers.

In conclusion, our findings did not show increased markers of oxidative stress and IL-8 in the airways of premature infants of mothers with hypertensive disorders of pregnancy in the first 72 h of life. Additionally, we found that these biomarkers were not predictive of BPD/death.
